# A conserved *EREG* ^+^ *IL1RN* ^+^ macrophage program defines an inflammation-associated transcriptional state linked to WTAP reprogramming and preterm premature rupture of membranes

**DOI:** 10.3389/fcell.2026.1903568

**Published:** 2026-10-28

**Authors:** Meng Zheng, Qiulan Yu, Xiaowei Zhang, Ping Yuan, Tongtong Liu, Haihong Wang

**Affiliations:** Department of Obstetrics and Gynecology, Binhai County People’s Hospital, Yancheng, Jiangsu, China

**Keywords:** EREG, IL1RN, inflammation, macrophages, preterm premature rupture of membranes, WTAP

## Abstract

Preterm premature rupture of membranes (PPROM) is a major cause of spontaneous preterm birth, yet the cellular mechanisms linking inflammation to membrane dysfunction remain incompletely understood. Here, we integrated single-cell and single-nucleus transcriptomic datasets from multiple maternal-fetal interface tissues to construct a comprehensive atlas of inflammatory signaling during early pregnancy. Macrophages emerged as the predominant source of basal inflammatory activity, and secondary clustering identified a conserved *EREG* ^+^ *IL1RN* ^+^ macrophages characterized by elevated inflammatory and innate immune programs. Pseudotime analysis revealed a transcriptional progression from proliferative and immunoregulatory macrophage states toward inflammatory and immune-effector states, with *EREG* ^+^ *IL1RN* ^+^ macrophages exhibiting a distinct transcriptional profile. Independent single-nucleus and spatial transcriptomic datasets confirmed the transcriptional and spatial conservation of this population. In placental infection datasets, *EREG* ^+^ *IL1RN* ^+^ macrophages were markedly expanded and enriched for cytokine signaling, leukocyte recruitment, and extracellular matrix remodeling pathways. *WTAP*, an m^6^A-associated candidate regulator, was positively associated with inflammatory macrophage programs, and its depletion attenuated inflammatory gene expression while promoting immunity hub-associated signatures. Clinically, the *EREG* ^+^ *IL1RN* ^+^ macrophage signature was significantly enriched in PPROM fetal membranes and showed the strongest ability to distinguish PPROM from preterm labor controls. Together, these findings identify *EREG* ^+^ *IL1RN* ^+^ macrophages as a conserved inflammatory macrophage program and implicate *WTAP* in inflammatory macrophage remodeling at the maternal-fetal interface.

## Background

Premature preterm rupture of membranes (PPROM) is a major obstetric complication that accounts for approximately one-third of spontaneous preterm births and remains a leading cause of neonatal morbidity and mortality worldwide (Goldenberg et al., 2008). Although advances in obstetric and neonatal care have improved clinical outcomes, the biological mechanisms underlying fetal membrane weakening and rupture remain incompletely understood (Menon and Richardson, 2017). Increasing evidence suggests that inflammation is a central driver of PPROM pathogenesis, linking maternal immune activation to extracellular matrix (ECM) degradation and structural failure of fetal membranes (Faro et al., 2019; Menon and Richardson, 2017). However, the cellular populations responsible for initiating and sustaining these inflammatory responses at the maternal-fetal interface remain poorly defined.

The maternal-fetal interface (MFI), comprising the decidua, placenta, fetal membranes, and associated immune compartments, represents a unique immunological environment in which fetal tolerance must coexist with effective antimicrobial defense (Li et al., 2020; Yang et al., 2019). Successful pregnancy depends on a tightly regulated balance between immune quiescence and controlled inflammatory activity (Li et al., 2020; Semmes and Coyne, 2022). While physiological inflammatory signaling contributes to tissue remodeling, vascular adaptation, and parturition, excessive or premature activation can trigger pathological cascades that culminate in membrane rupture and preterm birth (Faro et al., 2019; Menon and Richardson, 2017). Emerging evidence increasingly supports the concept that PPROM is not merely a localized tissue disorder but rather a manifestation of coordinated immune dysregulation across maternal and fetal compartments (Cappelletti et al., 2016; Lv et al., 2025; Menon and Taylor, 2019).

Among immune populations residing at the MFI, macrophages are particularly important regulators of tissue homeostasis and inflammatory responses (Duan et al., 2025; Semmes and Coyne, 2022). These cells exhibit remarkable functional plasticity and can dynamically transition between immunoregulatory, tissue-remodeling, and inflammatory states in response to environmental cues (Duan et al., 2025). Recent advances in single-cell transcriptomics have revealed extensive macrophage heterogeneity within placental and decidual tissues, challenging the traditional M1/M2 classification framework and uncovering specialized transcriptional states associated with pregnancy maintenance and disease (Levenson et al., 2025; Pique-Regi et al., 2019). In pathological contexts, inflammatory macrophages can establish feed-forward inflammatory circuits involving cytokine amplification, leukocyte recruitment, and matrix metalloproteinase activation, ultimately contributing to ECM degradation and membrane weakening (Gao et al., 2025; Menon et al., 2020). However, whether conserved macrophage programs exist across maternal-fetal tissues and how these states contribute to inflammatory amplification during PPROM remain largely unknown.

Recent advances in single-cell RNA sequencing have enabled systematic characterization of immune heterogeneity and intercellular communication within the maternal-fetal interface (Chen et al., 2022; Liu et al., 2024; Vento-Tormo et al., 2018; Wang W. S. et al., 2022; Zheng et al., 2025; Zhou et al., 2024). Integrative analyses across independent cohorts provide an unprecedented opportunity to identify conserved immune states, define their spatial organization, and uncover pathogenic programs associated with pregnancy complications. Such approaches are particularly valuable for understanding how inflammatory macrophage populations are established, maintained, and amplified under pathological conditions.

In addition to transcriptional regulation, emerging evidence suggests that epitranscriptomic mechanisms critically shape immune-cell function and inflammatory responses. N6-methyladenosine (m^6^A), the most abundant internal RNA modification in eukaryotic mRNAs, regulates transcript stability, translation efficiency, and cellular fate decisions (Leseva et al., 2023; Srivastava et al., 2025). The m^6^A methyltransferase complex, composed of METTL3, METTL14, and WTAP, coordinates deposition of m^6^A marks on target transcripts (Liu et al., 2014; Patil et al., 2016). Among these components, WTAP functions as an essential regulatory subunit that guides methyltransferase activity and facilitates RNA-protein interactions (Liu et al., 2014). Recent studies have demonstrated that WTAP-mediated m^6^A modification promotes inflammatory gene expression and macrophage activation in multiple pathological settings (Pinello et al., 2024), while m^6^A reader proteins further amplify inflammatory responses through selective recognition and translation of modified transcripts (Wang et al., 2015; Wang et al., 2024). However, whether m^6^A-dependent regulatory mechanisms govern macrophage state transitions at the maternal-fetal interface and contribute to PPROM-associated inflammation remains unknown.

In this study, we constructed an integrated single-cell atlas of the maternal-fetal interface by combining publicly available datasets from decidua, placenta, decidua basalis, myometrium, and peripheral blood. Through cross-dataset integration, spatial transcriptomic validation, and functional characterization, we identified a conserved *EREG* ^*+*^ *IL1RN* ^*+*^ macrophage program representing a prominent inflammatory macrophage state at the maternal-fetal interface. This macrophage program was reproducibly detected across independent cohorts, enriched under infectious inflammatory conditions, and associated with clinical PPROM. Furthermore, integrative analyses of m^6^A regulatory networks identified *WTAP* as a candidate regulator associated with inflammatory macrophage state transitions, and experimental perturbation supported its association with inflammatory macrophage polarization. Together, our findings establish *EREG* ^*+*^ *IL1RN* ^*+*^ macrophages as a conserved inflammatory macrophage program linked to maternal-fetal interface dysfunction and implicate *WTAP* in inflammatory macrophage remodeling in PPROM.

## Methods

### Data sources and sample acquisition

Publicly available single-cell and single-nucleus RNA sequencing datasets of early human pregnancy tissues were obtained from ArrayExpress and the European Genome-phenome Archive (EGA). These datasets provide spatially resolved multi-omic coverage of the placenta, decidua, decidua basalis, myometrium, and matched peripheral blood immune cells, enabling systematic investigation of immune heterogeneity and inflammatory programs at the maternal-fetal interface (Arutyunyan et al., 2023). The datasets analyzed in this study include E-MTAB-12421, E-MTAB-12595, EGAD00001010037, EGAD00001010038, EGAD00001010017, E-MTAB-12650, GSE198373, GSE212505 and GSE204684. These datasets correspond to tissues including blood, decidua, decidua basalis, and placenta.

To investigate pathogen-induced inflammatory responses, we additionally analyzed a published single-cell dataset of placental infection (E-MTAB-12795), which includes exposure to *Plasmodium falciparum*, *Listeria monocytogenes*, and *Toxoplasma gondii* (Hoo et al., 2024). Bulk RNA-seq datasets of fetal membranes from preterm labor (PTL) and preterm premature rupture of membranes (PPROM) were further incorporated to validate macrophage-associated inflammatory programs. In the PPROM/PTL dataset, Amnion and Chorion tissues were analyzed separately with n = 4 per group, corresponding to GSE243831.

### Single-cell data processing and integration

Raw count matrices were processed using the Seurat R package (v4.0 or later)(Hao et al., 2021). Cells with low-quality metrics (mitochondrial gene percentage >10% or fewer than 200 detected genes) were excluded. Data were log-normalized, and highly variable genes were identified using the FindVariableFeatures function.

Principal component analysis (PCA) was performed on scaled expression data, and the top 30 principal components were used for downstream analyses. To integrate datasets and mitigate donor- and tissue-specific batch effects, Harmony was applied to the PCA embeddings with donor identity as the batch variable (Korsunsky et al., 2019).

The integrated embeddings were visualized using Uniform Manifold Approximation and Projection (UMAP) with parameters n_neighbors = 30 and min_dist = 0.3. Nearest-neighbor graphs were constructed using the FindNeighbors function, followed by clustering using FindClusters with a resolution of 0.5 unless otherwise specified (Hao et al., 2021).

### Cell-type annotation

Cell clusters were annotated based on canonical marker gene expression and differential expression analysis using the FindAllMarkers function (min.pct = 0.25, log fold change threshold = 0.25)(Hao et al., 2021). Reference gene signatures from published single-cell atlases were incorporated to improve annotation accuracy. Low-quality or ambiguous clusters were excluded from downstream analyses (Vento-Tormo et al., 2018; Wang Q. et al., 2022). Annotation confidence was cross-validated across independent datasets to ensure robustness.

### Macrophage sub-clustering

Macrophage populations were subsetted based on canonical myeloid marker genes. The extracted cells were re-normalized, scaled, and subjected to PCA, UMAP, and clustering to identify transcriptionally distinct subsets (Macro_1-Macro_5). Differential gene expression analysis between subsets was performed using the FindMarkers function with the Wilcoxon rank-sum test to identify subset-associated transcriptional features. *P*-values were adjusted using the Benjamini–Hochberg method.

Module scores representing inflammation, Th17 signaling, neutrophil recruitment, extracellular matrix (ECM) remodeling, and immune activation were calculated using the AddModuleScore function. Gene sets were curated from the literature or derived from subset-specific differentially expressed genes, and control gene sets were selected to match expression distributions. These module scores were used to characterize functional program activity at the single-cell level.

### Cross-dataset integration and subset conservation

To assess conservation of macrophage programs, independent infection and clinical PPROM/PTL datasets were integrated using the same Harmony-based workflow. Macrophages were re-clustered, and transcriptional correspondence with *EREG* ^*+*^ *IL1RN* ^*+*^ macrophages was assessed via module scoring and correlation of subset-specific gene expression. Proportion and activation levels of conserved inflammatory macrophage subsets were compared across physiological, infection, and pathological conditions.

For the bulk RNA-seq analysis of PPROM/PTL fetal membranes, macrophage-specific gene signatures corresponding to M_1-M_3 programs were used to calculate relative module scores in Amnion and Chorion samples. Sample classification into PPROM or PTL groups was performed according to GSM IDs. Receiver operating characteristic (ROC) curves and area under the curve (AUC) were calculated using the pROC R package to exploratorily assess the discriminatory ability of M_1-M_3 programs.

### Functional enrichment analysis

Differentially expressed genes (adjusted P < 0.05, log fold change >0.25) were subjected to Gene Ontology (GO) and Kyoto Encyclopedia of Genes and Genomes (KEGG) enrichment analyses using the clusterProfiler R package (Ashburner et al., 2000; Kanehisa and Goto, 2000; Yu et al., 2012). Significantly enriched biological processes and pathways were identified based on adjusted P values and visualized using bar plots. Functional modules were further quantified using module scoring across cell types and conditions. Curated gene sets for inflammatory response, Th17 signaling, neutrophil recruitment, ECM remodeling, and immune regulation were used to calculate module scores.

### Visualization

UMAP plots, heatmaps, violin plots, and dot plots were generated using Seurat (Butler et al., 2018) and ggplot2. Gene expression values were log-normalized (ln [1 + x]) and scaled to zero mean and unit variance for heatmap visualization. Module scores were visualized using violin plots or feature plots to illustrate functional differences across cell types, macrophage subsets, and experimental conditions.

Inflammation scores were calculated based on curated sets of inflammation-related genes, summarized as averaged module scores per cell. These module scores were used for descriptive visualization of the cellular distribution of inflammation-related transcriptional programs.

### m^6^A regulator compilation

To characterize m^6^A-related regulatory programs, a panel of 20 established m^6^A regulators was assembled based on published literature and functional annotations. The panel comprised methyltransferase components and cofactors (*METTL3*, *METTL14*, *WTAP*, *RBM15*, *RBM15B*, and *ZC3H13*), demethylases (*FTO* and *ALKBH5*), and m^6^A-binding proteins (*YTHDF1*, *YTHDF2*, *YTHDF3*, *YTHDC1*, *YTHDC2*, *IGF2BP1*, *IGF2BP2*, *IGF2BP3*, *FMR1*, *LRPPRC*, *ELAVL1*, and *CBLL1*). Gene identifiers were harmonized according to the approved HGNC nomenclature prior to downstream analyses.

### Pathway activity quantification and correlation analysis

Pathway activities were quantified at the single-cell level using the AddModuleScore function implemented in the Seurat package. Signature gene sets representing inflammatory responses, immune regulation, and immunity-associated pathways were collected from previously published studies. Module scores were calculated from log-normalized expression matrices and subsequently standardized within individual cell populations to facilitate cross-cell-type comparisons. To explore the relationship between m^6^A regulators and biological pathways, Spearman rank correlation analyses were performed between regulator expression levels and pathway activity scores within each annotated cell type. Statistical significance was assessed using correlation tests, followed by Benjamini–Hochberg correction for multiple comparisons. Correlation coefficients together with adjusted *P* values were used for downstream interpretation and visualization.

### Generation of PBMC-derived macrophages and gene silencing

Peripheral blood mononuclear cells were commercially obtained from Beijing Mumi Xing Biotechnology Co., Ltd. (Leuko Pak, Cat. No. PB007-2) from 4 independent donor lots (LP240918A, LP241030C, LP241114E, and LP241120A). Monocytes were enriched by plastic adherence and cultured in RPMI-1640 medium supplemented with 10% fetal bovine serum and macrophage colony-stimulating factor (M-CSF; CHAMOT Biotechnology, Cat# CM061-20 MP) to induce macrophage differentiation. After 6–7 days of culture, mature macrophages were collected for subsequent experiments.

For transient knockdown experiments, macrophages were transfected with WTAP-specific siRNA or a scrambled control oligonucleotide using Lipofectamine™ 2000 (Invitrogen) according to the manufacturer’s instructions. Synthetic siRNA duplexes were obtained from Sangon Biotech (Shanghai, China). Following a 6-h transfection period, the transfection medium was replaced with fresh complete medium, and cells were allowed to recover before inflammatory stimulation and downstream functional assays.

For intracellular WTAP staining, macrophages were fixed, permeabilized, and stained with anti-WTAP antibody (ab195380; Abcam) according to the manufacturer’s instructions, followed by Goat Anti-Rabbit IgG Alexa Fluor™ 594 (A-11037; Thermo Fisher Scientific) as the secondary antibody. Flow cytometry was performed on a BD LSRFortessa and analyzed using FlowJo v10. Cells were sequentially gated based on FSC/SSC, singlets, live cells, macrophages, and WTAP positivity. A fluorescence-minus-one (FMO) control was used to define the threshold for WTAP positivity, and the percentage of WTAP^+^ macrophages was quantified for independent donor sample.

### Quantitative real-time PCR

Total RNA was extracted from cultured macrophages using TRIzol reagent (Invitrogen). First-strand cDNA synthesis was performed with TRUEscript RT Master Mix (Aidlab, Cat# PC58). Quantitative PCR amplification was carried out using 2× SYBR Green qPCR Mix (Aidlab, Cat# PC3301) on a real-time PCR platform according to the manufacturer’s recommendations. Relative transcript abundance was determined using the comparative Ct (2^−ΔΔCt^) method. β-actin served as endogenous reference gene for normalization. Primer sequences used for amplification are provided in Supplementary Table S9.

### Quantification and statistical analysis

Statistical analyses were carried out using GraphPad Prism (version 8.3.0), FlowJo (version 10.10.2), and R software (version 4.2.1). For pairwise comparisons, two-tailed paired or unpaired Student’s t-tests were applied as appropriate; for paired comparisons, samples derived from the same donor were treated as paired observations. For qPCR analyses, statistical comparisons were performed using relative expression values calculated by the 2^−ΔΔCt^ method. All experiments included biological replicates, and the exact number of samples (n) as well as the statistical methods used for each analysis are detailed in the corresponding figure legends. For comparisons between biological conditions, independent donors or biological samples were considered the experimental units, whereas individual cells were used to characterize cellular heterogeneity. Data are reported as mean ± standard deviation (SD) unless otherwise specified.

Statistical significance was defined using standard notation as follows: n. s., not significant; *, *P* < 0.05; **, *P* < 0.01; ***, *P* < 0.001; ****, *P* < 0.0001.

## Results

### An integrated single-cell atlas reveals tissue-specific inflammatory programs and macrophage-dominated basal inflammatory tone at the maternal-fetal interface

To systematically characterize cellular composition and inflammatory signaling at the maternal-fetal interface under homeostasis, we integrated publicly available single-cell and single-nucleus RNA-seq datasets from early human pregnancy tissues, including placenta, decidua, decidua basalis, and myometrium, together with peripheral blood immune cells (Supplementary Table S1). The resulting atlas captured a broad spectrum of immune and stromal populations, providing a cross-tissue framework for dissecting maternal-fetal inflammation and its potential pathological perturbations. All datasets underwent uniform quality control and normalization. Cells from different tissues were aggregated and batch-corrected using Harmony in PCA space to minimize donor- and tissue-specific biases. The integrated map showed clear separation of major cell types in low-dimensional space, with clustering largely driven by lineage identity rather than tissue origin (Figure 1A). Canonical marker analysis confirmed the presence of immune and stromal cell populations. Each cell type exhibited stable and specific marker expression, supporting the biological fidelity of the atlas and providing a reliable basis for downstream analyses (Figure 1B).

**FIGURE 1:**
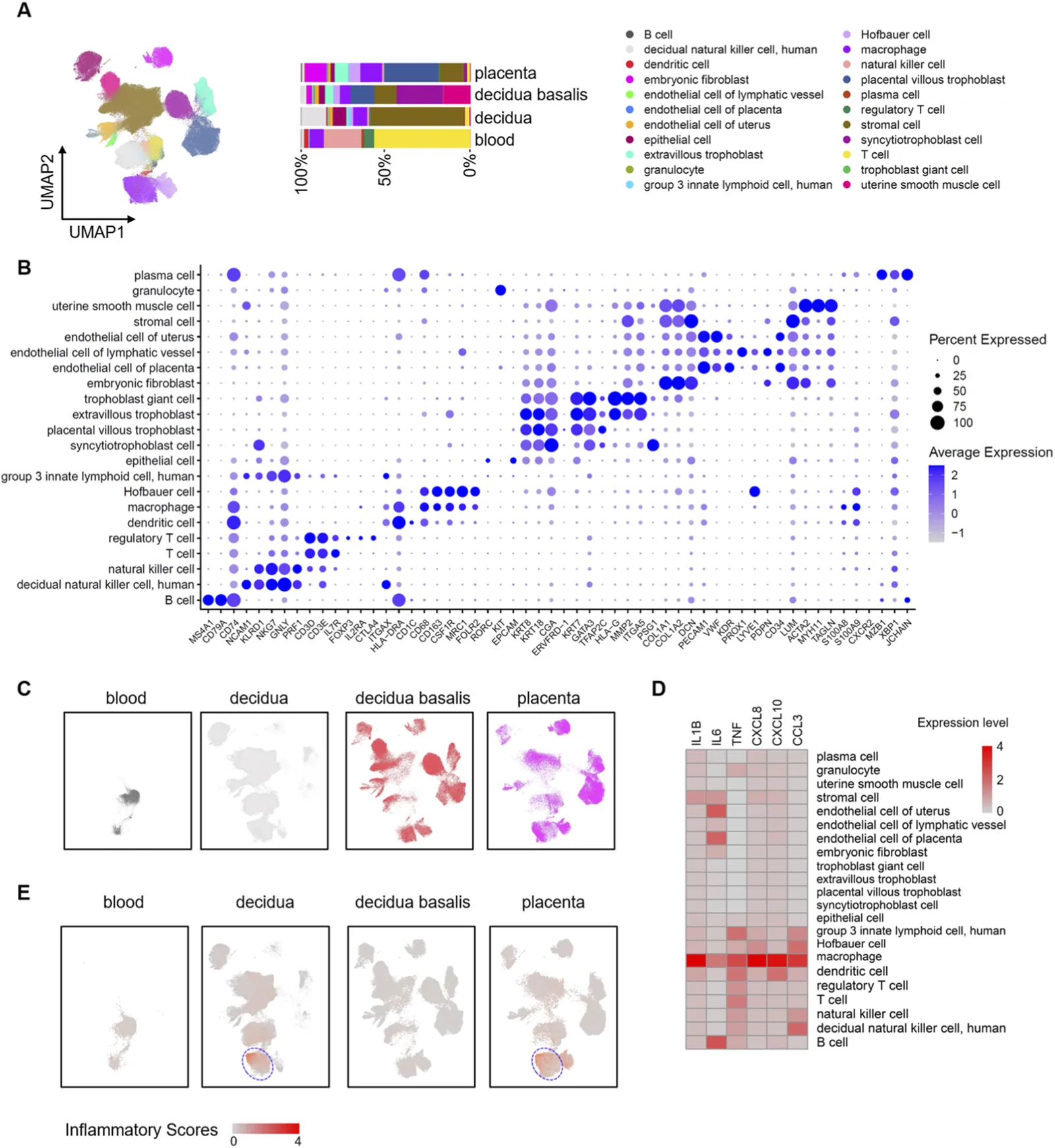
Integrated single-nucleus atlas reveals tissue-specific inflammatory programs across the maternal-fetal interface and peripheral blood. **(A)** UMAP projection of integrated single-nucleus transcriptomic data following Harmony-based correction of principal component analysis (PCA) embeddings. Cells are colored according to their annotated cell identities. Donor demographic information for matched blood, decidua, decidua basalis, and placenta samples is summarized in Supplementary Table S1. **(B)** DotPlot showing the scaled expression of representative marker genes across different cell types in **(A)**. **(C)** UMAP visualization of cells derived from healthy donors, stratified and colored by tissue of origin, including blood, decidua, decidua basalis, and placenta, highlighting tissue-specific cellular distribution patterns. **(D)** Heatmap illustrating the scaled expression of representative inflammatory genes across the annotated cell populations shown in **(A)**. **(E)** UMAP plots of all cells from blood, decidua, decidua basalis, and placenta, with each cell colored by its inflammatory score.

Next, we analyzed the cell composition across distinct tissue types. T cells and NK cells were predominantly derived from peripheral blood (Figures 1A,C). Stromal cells were most abundant in decidua. Syncytiotrophoblasts were primarily derived from decidua basalis (Figures 1A,C). In contrast, macrophages were broadly distributed across multiple tissues, suggesting their role as key immune mediators spanning different maternal-fetal interface regions (Figures 1A,C; Supplementary Table S2). To delineate the primary cellular contributors to inflammation, we systematically analyzed the expression of key inflammatory genes-including *IL1B*, *IL6*, *TNF*, *CXCL8*, *CXCL10*, and *CCL3*-across distinct cell populations. Results showed the expression of key inflammation-related genes was predominantly restricted to macrophages, whereas other immune and stromal populations exhibited minimal expression. (Figure 1D; Supplementary Table S3). Stratifying by tissue origin revealed that decidua- and placenta-derived macrophages exhibited the strongest inflammatory signatures, while peripheral blood macrophages had limited expression (Figure 1E). These results indicate that tissue-resident macrophages at the maternal-fetal interface are the primary source of basal inflammatory signaling, highlighting their prominent role in local immune activity.

### Macrophage sub-clustering identifies a *EREG* ^*+*^ *IL1RN* ^*+*^ macrophage subset associated with amplified inflammation at the maternal-fetal interface

Recent single-cell transcriptomic studies have highlighted the remarkable plasticity and functional heterogeneity of macrophages across tissues under both homeostatic and pathological conditions (Dominguez Conde et al., 2022; Garrido-Trigo et al., 2023; Hume et al., 2023). However, the identity and organization of macrophage subsets that drive inflammatory amplification at the maternal-fetal interface remain incompletely defined. To address this, we integrated single-cell atlases and identified macrophages as a major source of basal inflammatory signaling within the maternal-fetal niche. We therefore performed secondary clustering to resolve macrophage heterogeneity at higher resolution.

Unsupervised re-clustering based on highly variable genes and low-dimensional embeddings identified five transcriptionally distinct macrophage subsets (Macro_1-Macro_5) (Figure 2A; Supplementary Table S4). Tissue distribution analysis revealed distinct spatial preferences, with Macro_1 detected in both decidua and placenta, Macro_2 enriched in placenta, Macro_3 and Macro_4 predominantly restricted to decidua, and Macro_5 largely confined to peripheral blood. These findings suggest compartment-specific specialization of macrophage states across the maternal-fetal interface.

**FIGURE 2:**
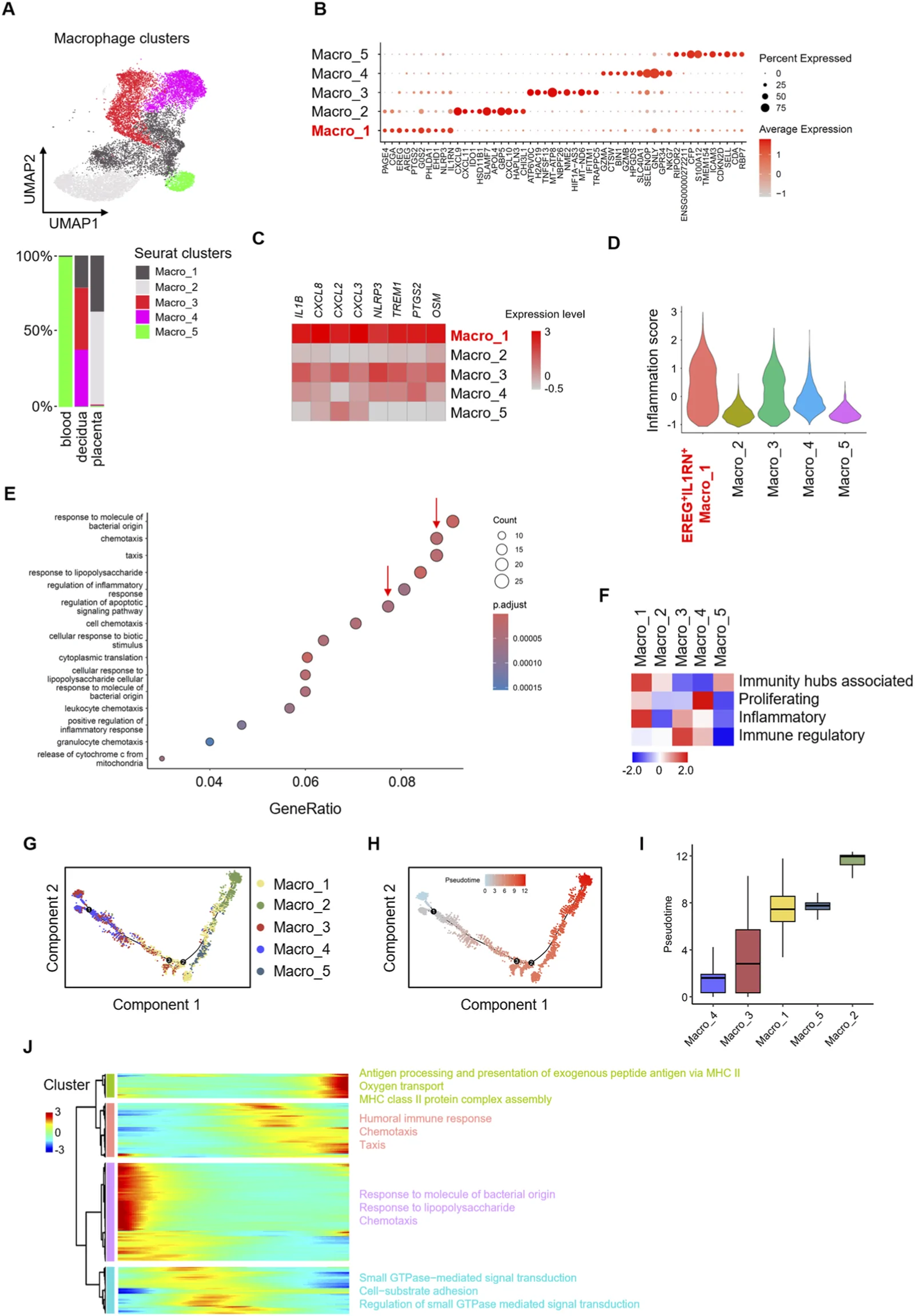
*EREG* ^*+*^ *IL1RN* ^*+*^ macrophages mark a pro-inflammatory subset with enriched immune activation pathways. **(A)** UMAP projection of re-clustered macrophages, identifying five distinct subsets (Macro_1–Macro_5) (upper panel). Stacked bar plot showing the relative abundance of each macrophage subset across different tissue types (lower panel). **(B)** Dot plot showing the scaled expression levels of the top 10 marker genes defining each macrophage subset. **(C)** Heatmap illustrating the expression profiles of representative inflammatory genes across the macrophage subsets identified in **(A)**. **(D)** Violin plots displaying inflammatory signature scores across distinct macrophage subsets. Scores are shown at the single-cell level to characterize cellular heterogeneity. **(E)** Gene Ontology (GO) enrichment analysis of differentially expressed genes (DEGs) in Macro_1 (*EREG* ^*+*^ *IL1RN* ^*+*^ macrophages). The bar plot presents the top 15 significantly enriched biological process terms. **(F)** Heatmap showing the expression of genes associated with immune regulation, inflammatory responses, proliferative programs, and immunity hub-related signatures across macrophage subsets. **(G,H)** Monocle2 pseudotime analysis showing the inferred transcriptional ordering of macrophage states using Macro_4 as the computational root **(G)** and illustrating the corresponding pseudotime progression **(H)**. **(I)** Boxplot showing the distribution of pseudotime values among macrophage subsets, reflecting their relative transcriptional ordering. **(J)** Heatmap depicting dynamic gene expression changes along the pseudotime trajectory. Differentially expressed genes were hierarchically clustered into three distinct modules, and representative biological pathways enriched within each module are indicated.

Differential gene expression analysis further indicated that these subsets are associated with distinct functional programs (Figures 2A,B). Among them, Macro_1 displayed a coordinated inflammatory transcriptional signature characterized by concurrent expression of *PTGS2*, *NLRP3*, *IL1RN*, and *EREG*, together with additional immune activation markers, suggesting a poised inflammatory state with regulatory features. Macro_2 was enriched for interferon-responsive chemokines (*CXCL9*, *CXCL10*, *CXCL11*) and immunoregulatory genes including IDO1, consistent with an inflammation-responsive phenotype linked to immune cell recruitment. Macro_3 exhibited enrichment of metabolic and stress-associated transcripts, whereas Macro_4 was characterized by expression of immune interaction-associated genes including *GZMB* and *NKG7*, suggesting an activated immune-interfacing state within the decidua. Macro_5 showed a circulating signature marked by adhesion and trafficking-related genes such as *SELL* and *ICAM3*, consistent with a peripheral blood-derived population. Together, these results indicate that macrophage heterogeneity at the maternal-fetal interface is structured along both tissue origin and functional specialization.

Among these subsets, Macro_1 exhibited the most prominent enrichment of inflammatory and immune activation genes (Figure 2C; Supplementary Table S5), and was therefore defined as the *EREG* ^*+*^ *IL1RN* ^*+*^ macrophages. Quantitative module scoring confirmed that this subset displayed higher inflammatory activity compared with other macrophage states (Figure 2D), indicating a persistently activated inflammatory phenotype. Functional enrichment analysis of *EREG* ^*+*^ *IL1RN* ^*+*^ macrophage signature genes revealed strong involvement in innate immune sensing pathways, including responses to bacterial-derived molecules and lipopolysaccharide, as well as chemotaxis and leukocyte recruitment programs (Figure 2E). These pathways were further associated with regulation of apoptotic signaling and protein synthesis, suggesting an integrated inflammatory and effector response capacity.

Pseudotime trajectory analysis, with the proliferative Macro_4 state selected as the computational root based on its prominent cell-cycle and proliferation-associated gene expression, revealed a continuous transcriptional trajectory connecting proliferative (Macro_4) and immunoregulatory (Macro_3) states with inflammatory and immune effector-associated states (Macro_1, Macro_2, and Macro_5) (Figures 2F–I). This trajectory suggests a continuous transition in macrophage transcriptional states accompanied by changes in inflammatory and immune-activating programs. To further delineate the molecular programs underlying this transcriptional continuum, GO enrichment analysis was performed across pseudotime-defined transcriptional stages (Figure 2J). Early-stage macrophages were enriched in innate immune sensing pathways, including responses to bacterial components and lipopolysaccharide, together with chemotactic processes. Intermediate states were characterized by enrichment of cytoskeletal remodeling and small GTPase-mediated signaling, consistent with dynamic changes in migration and cell-matrix interactions. Late-stage macrophages displayed enrichment of antigen processing and presentation via MHC class II, MHC II complex assembly, and humoral immune response pathways, indicating a transition toward antigen-presenting and functionally specialized immune states.

Collectively, these results define a structured macrophage transcriptional state landscape at the maternal-fetal interface, linking tissue localization with variation in inflammatory and antigen-presenting programs across the inferred transcriptional continuum.

### Cross-dataset integration identifies a conserved *EREG* ^*+*^ *IL1RN* ^*+*^ macrophages

To validate the robustness and cross-dataset conservation of the EREG^+^IL1RN^+^ macrophages, we first analyzed an independent single-nucleus RNA-seq dataset encompassing 33 annotated placental cell types (Figure 3A). UMAP visualization demonstrated a clear separation of major cellular compartments across the maternal-fetal interface. Within this integrated atlas, both *EREG* and *IL1RN* were predominantly enriched in macrophage populations, as revealed by feature expression mapping on the UMAP space (Figure 3B). Notably, their expression was largely restricted to a specific macrophage compartment rather than being broadly distributed across immune or stromal lineages. This observation was further supported by dot plot analysis, which showed that *EREG* and *IL1RN* expression was specifically enriched in *CD16* ^*+*^ macrophage subsets, indicating that this transcriptional signature defines a distinct macrophage state at the mRNA level (Figure 3C). These findings collectively support the existence of a conserved *EREG* ^*+*^ *IL1RN* ^*+*^ macrophages across independent single-nucleus datasets.

**FIGURE 3:**
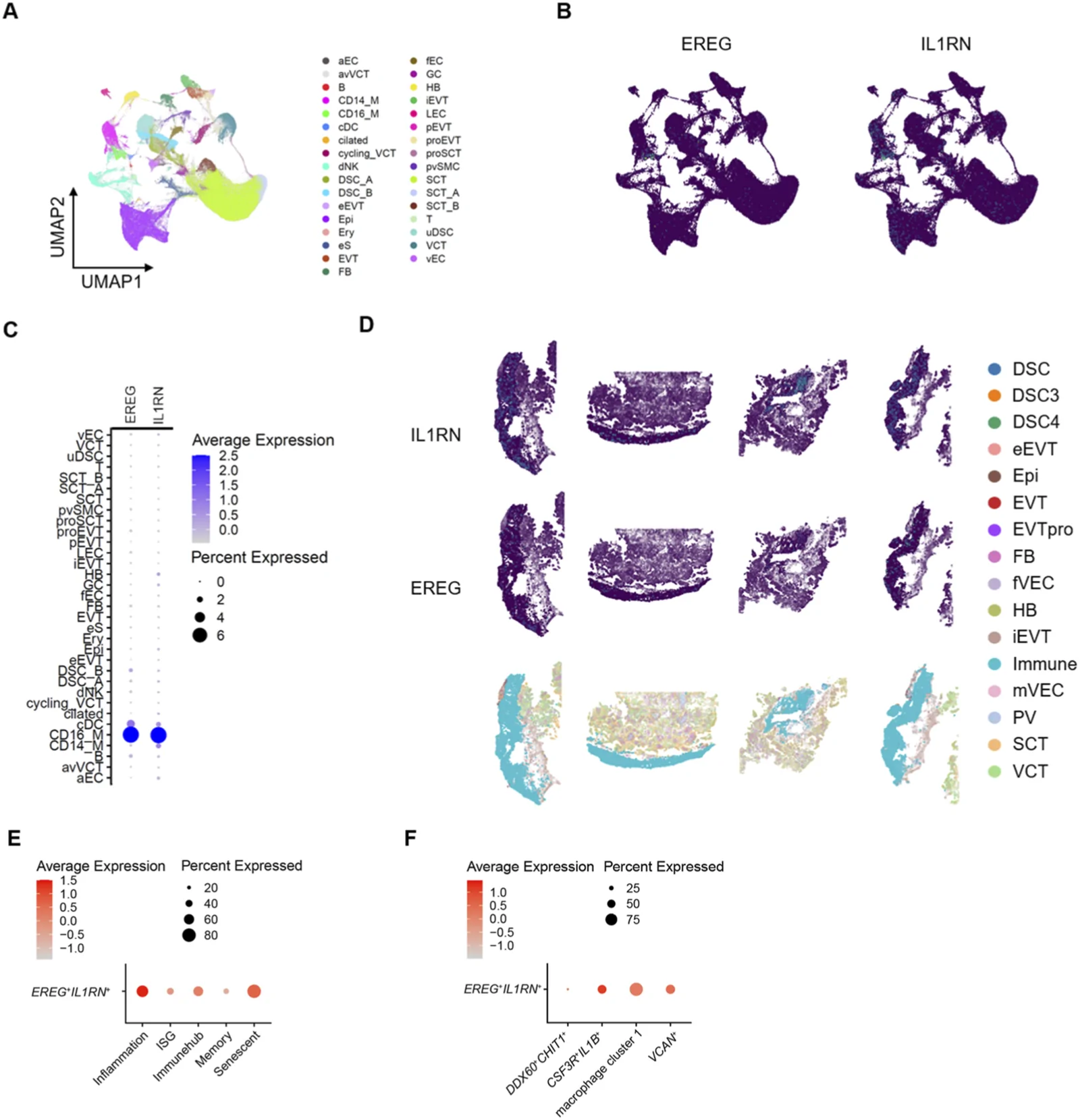
Cross-dataset integration validates the conservation of the *EREG* ^*+*^ *IL1RN* ^*+*^ macrophages. **(A)** UMAP projections of snRNA-seq for 33 broadly identified cell types. Cells are colored according to their annotated cell identities. **(B)** UMAP showing the expression of *EREG* and *IL1RN* across different cell types in **(A)**. **(C)** DotPlot showing the expression of *EREG* and *IL1RN* across different cell types in **(A)**. **(D)** Spatial distribution of *EREG*- and *IL1RN*-positive cells in the human basal plate identified by Stereo-seq spatial whole-transcriptome sequencing. **(E)** Enrichment scores of established macrophage functional states in the *EREG* ^+^ *IL1RN* ^+^ macrophages. **(F)** Enrichment scores of previously reported inflammatory macrophage signatures in the *EREG* ^+^ *IL1RN* ^+^ macrophages. Abbreviations of cell populations shown in panels **(A)** and **(D)** aVCT, anchoring villi cytotrophoblast; CD14_M, CD14-positive macrophage; CD16_M, CD16-positive macrophage; cEVT, column extravillous trophoblast; cycling_VCT, cycling villous cytotrophoblast; DSC, decidual stromal cell; DSC_A/B, decidual stromal cell subtype A/B; Epi, epithelial cell; Ery, erythroid cell; EVT, extravillous trophoblast; FB, fibroblast; GC, glandular cell; HB, hemangioblast; iEVT, invasive extravillous trophoblast; Immune, immune cell; pEVT, progressive extravillous trophoblast; preSCT, precursor spiral artery cytotrophoblast; PV, perivascular cell; SCT, spiral artery cytotrophoblast; T, T cell; uDSC, uterine decidual stromal cell; vEC, vascular endothelial cell; VCT, villous cytotrophoblast; EVTpro, extravillous trophoblast progenitor; and mVEC, maternal vascular endothelial cell.

To further assess spatial consistency at the tissue level, we interrogated Stereo-seq spatial transcriptomic data from the human placental basal plate. Spatial mapping revealed co-localization of *EREG* and *IL1RN* expression, with both signals preferentially enriched in immune cell-rich regions of the maternal-fetal interface (Figure 3D). This spatial co-distribution further supports the conservation of the *EREG* ^*+*^ *IL1RN* ^*+*^ macrophages across transcriptomic and spatially resolved datasets. To further define its relationship with previously described macrophage states, we assessed enrichment scores for established macrophage functional programs (Figure 3E). The *EREG* ^+^ *IL1RN* ^+^ population showed prominent enrichment of inflammatory macrophage features, together with a degree of senescence-like characteristics. Consistently, comparison with previously reported inflammatory macrophage signatures revealed varying degrees of enrichment across multiple inflammatory macrophage states (Figure 3F). Collectively, these multi-modal analyses demonstrate that *EREG* ^*+*^ *IL1RN* ^*+*^ macrophages represent a conserved inflammatory transcriptional state with features overlapping established macrophage programs, rather than a completely distinct macrophage lineage, at the maternal-fetal interface.

### *EREG* ^*+*^ *IL1RN* ^*+*^ macrophages exhibit cross-cohort robustness and dynamic activation in pathology

Building on the integrated maternal-fetal interface atlas, we identified a conserved macrophage subset characterized by a coordinated inflammatory program, defined as *EREG* ^*+*^ *IL1RN* ^*+*^ macrophages. To further assess the robustness of this population across independent cohorts and its behavior under pathological inflammatory stimulation, we analyzed a publicly available single-cell transcriptomic dataset of early placental infections, encompassing responses to *P. falciparum*, *Listeria* monocytogenes, and *T. gondii* (Hoo et al., 2024). This dataset provides a comprehensive framework for evaluating pathogen-induced immune remodeling in placental macrophages.

Following data integration, dimensionality reduction, and cell-type annotation, major immune populations were consistently resolved across infection conditions, indicating preserved cellular identities despite strong inflammatory perturbation (Figures 4A,B). Notably, macrophages exhibited broad enrichment of inflammatory programs across all infection contexts, supporting their central role as primary mediators of placental immune activation (Figures 4C,D; Supplementary Table S6). To determine whether infection-associated inflammatory states recapitulate the *EREG* ^*+*^ *IL1RN* ^*+*^ macrophage program, we performed secondary clustering of macrophages within the independent dataset, identifying three transcriptionally distinct subsets (M_1-M_3) (Figure 4E; Supplementary Table S7). Among these, the M_3 subset displayed a transcriptional signature highly consistent with the *EREG* ^*+*^ *IL1RN* ^*+*^ macrophages identified at the maternal-fetal interface, characterized by elevated expression of inflammatory mediators and immune activation-related genes (Figure 4F; Supplementary Table S8). Importantly, M_3 cells were significantly enriched under pathogen exposure conditions (Figure 4G), indicating that this conserved macrophage state is dynamically amplified during infection. These results demonstrate that *EREG* ^*+*^ *IL1RN* ^*+*^ macrophages represent a stable inflammatory macrophage program across independent cohorts, which can be further induced under pathological conditions. This supports a model in which a conserved basal inflammatory macrophage state at the maternal-fetal interface becomes transcriptionally amplified in response to infectious stimuli, reflecting a “baseline-to-pathological activation” continuum.

**FIGURE 4:**
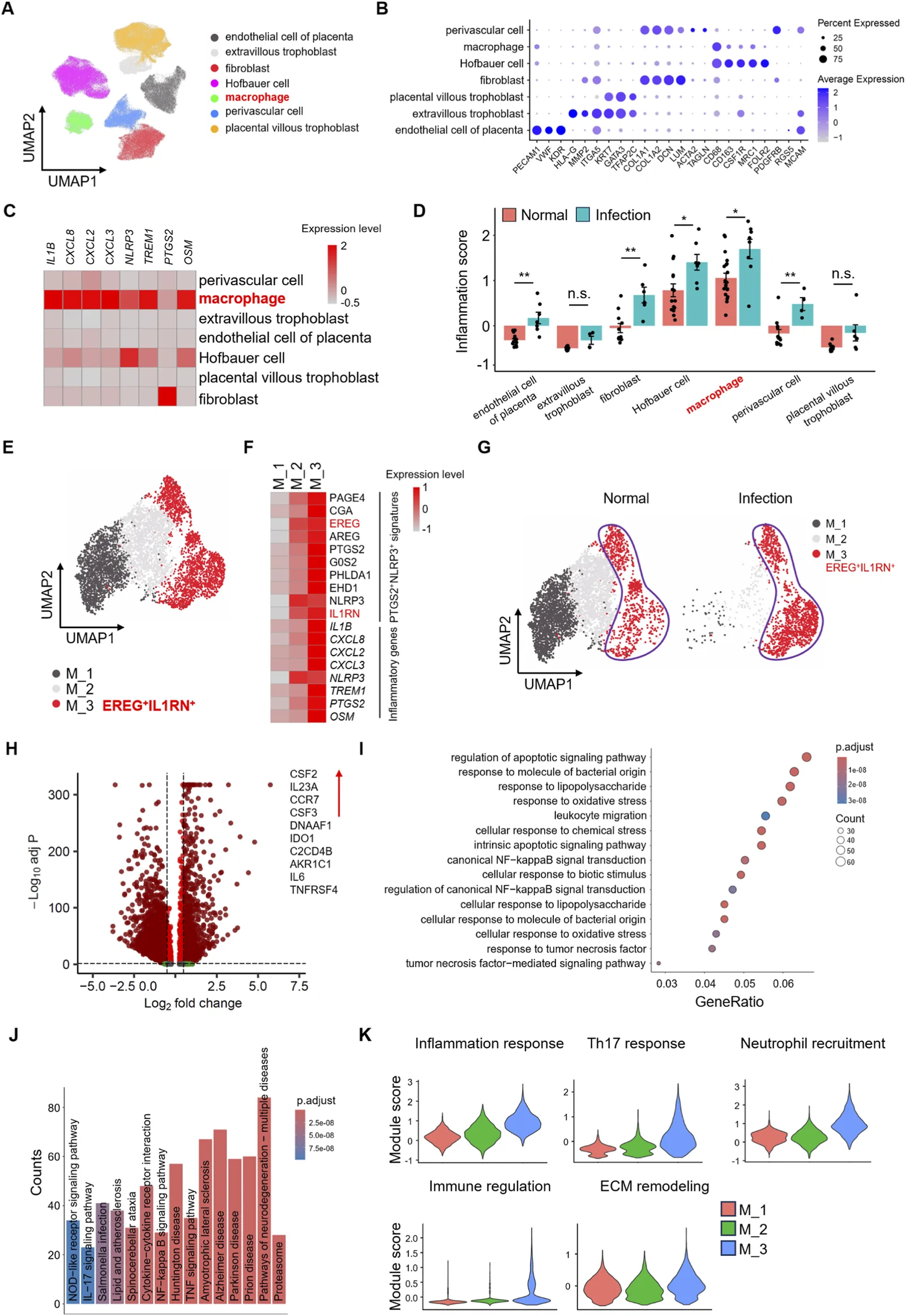
The *EREG* ^*+*^ *IL1RN* ^*+*^ macrophage subset exhibits a pro-inflammatory and tissue-remodeling phenotype in infected placentas. **(A)** UMAP projection of integrated single-cell transcriptomic data from a publicly available dataset of acute infections in early human placenta, following Harmony-based correction of principal component analysis (PCA) embeddings. Cells are colored according to their annotated cell identities. **(B)** DotPlot showing the scaled expression of representative marker genes across different cell types. **(C)** Heatmap showing the scaled expression of inflammation-related genes across the annotated cell types identified in **(A)**. **(D)** Violin plots showing inflammation scores across different cell types in normal (n = 10–20) and infection (n = 4–8) groups, with the number of independent biological samples (donors) varying across cell types. Statistical comparisons were performed using individual biological samples as the experimental units. **(E)** UMAP visualization of re-clustered macrophages, annotated as M_1-M_3. **(F)** Heatmap showing the *EREG* ^*+*^ *IL1RN* ^*+*^ macrophage gene signature scores and expression of inflammatory genes across macrophage subsets. **(G)** UMAP projection of macrophages colored by experimental group (normal versus infection), highlighting group-specific distribution patterns. **(H)** Volcano plot showing differential gene expression in the M_3 (*EREG* ^*+*^ *IL1RN* ^*+*^) macrophage subset. Significantly upregulated genes are highlighted, with the top 10 most upregulated genes. **(I)** GO enrichment analysis of marker genes for the M_3 subset. Bar plot displaying the top 15 significantly enriched biological process terms. **(J)** KEGG pathway enrichment analysis of marker genes for the M_3 subset. Bar plot displaying the top 15 significantly enriched pathways. **(K)** Violin plots showing the distribution of module scores for inflammatory response, Th17 response, neutrophil recruitment, immune regulation, and extracellular matrix (ECM) remodeling across macrophage subsets. Scores are shown at the single-cell level to characterize cellular heterogeneity. n. s., no significance; *, *P* < 0.05; **, *P* < 0.01 (two-tailed unpaired Students’ t-test in **(D)**.

To further define the functional properties of this conserved state, we next examined genes upregulated in the M_3 subset. Differential expression analysis revealed enrichment of canonical inflammatory mediators, including cytokines, chemokines, and immune activation-associated genes (Figure 4H), indicating a highly activated inflammatory phenotype. Functional enrichment analysis further demonstrated that M_3-associated genes were significantly enriched in pathways involved in cytokine production, leukocyte migration, regulation of inflammatory responses, and sensing of bacterial components such as *lipopolysaccharide* (Figure 4I). Additional enrichment of NF-κB signaling, TNF signaling, and apoptotic pathways suggested integration of pathogen sensing with downstream inflammatory and stress-response programs. Consistently, KEGG pathway analysis highlighted activation of NOD-like receptor, IL-17, NF-κB, and TNF signaling pathways, as well as cytokine-cytokine receptor interactions (Figure 4J).

Finally, module scoring across predefined immune and tissue functional signatures revealed that *EREG* ^*+*^ *IL1RN* ^*+*^ macrophages exhibited higher activity in inflammatory response, Th17-associated signaling, neutrophil recruitment, immune regulation, and extracellular matrix (ECM) remodeling programs (Figure 4K). Notably, concurrent activation of neutrophil recruitment and Th17-related pathways suggests a role for this subset in amplifying innate immune cascades, whereas enrichment of ECM remodeling signatures indicates potential involvement in tissue structural modulation under inflammatory conditions.

Collectively, these findings establish *EREG* ^*+*^ *IL1RN* ^*+*^ macrophages as a conserved inflammatory macrophage program across independent datasets, capable of robust activation under infection, and positioned as a central effector population linking immune amplification and tissue remodeling at the maternal-fetal interface.

### *WTAP* is associated with inflammatory reprogramming of *EREG* ^*+*^ *IL1RN* ^*+*^ macrophages during state transitions

To investigate whether epitranscriptomic regulation contributes to macrophage state transitions between immunity hub-associated and inflammatory programs, we focused on N6-methyladenosine (m^6^A), the most abundant internal mRNA modification. m^6^A regulatory factors modulate RNA stability and translational efficiency, thereby shaping immune activation and cellular state dynamics. However, whether these regulatory programs primarily reflect lineage identity or functional macrophage states within the maternal-fetal microenvironment remains unclear. To address this, we first performed unsupervised clustering based on global transcriptomic profiles, which separated macrophages into distinct functional compartments. Proliferative and immunoregulatory states (Macro_3 and Macro_4) clustered together, whereas inflammatory and immunity hub–associated states (Macro_1, Macro_2, and Macro_5) formed a separate branch (Figure 5A). We next reconstructed macrophage organization using only m^6^A regulator expression. Notably, m^6^A-based clustering partially recapitulated the structure observed in transcriptome-based clustering, suggesting that m^6^A regulatory signatures are associated with macrophage functional states rather than strict lineage identity (Figure 5B).

**FIGURE 5:**
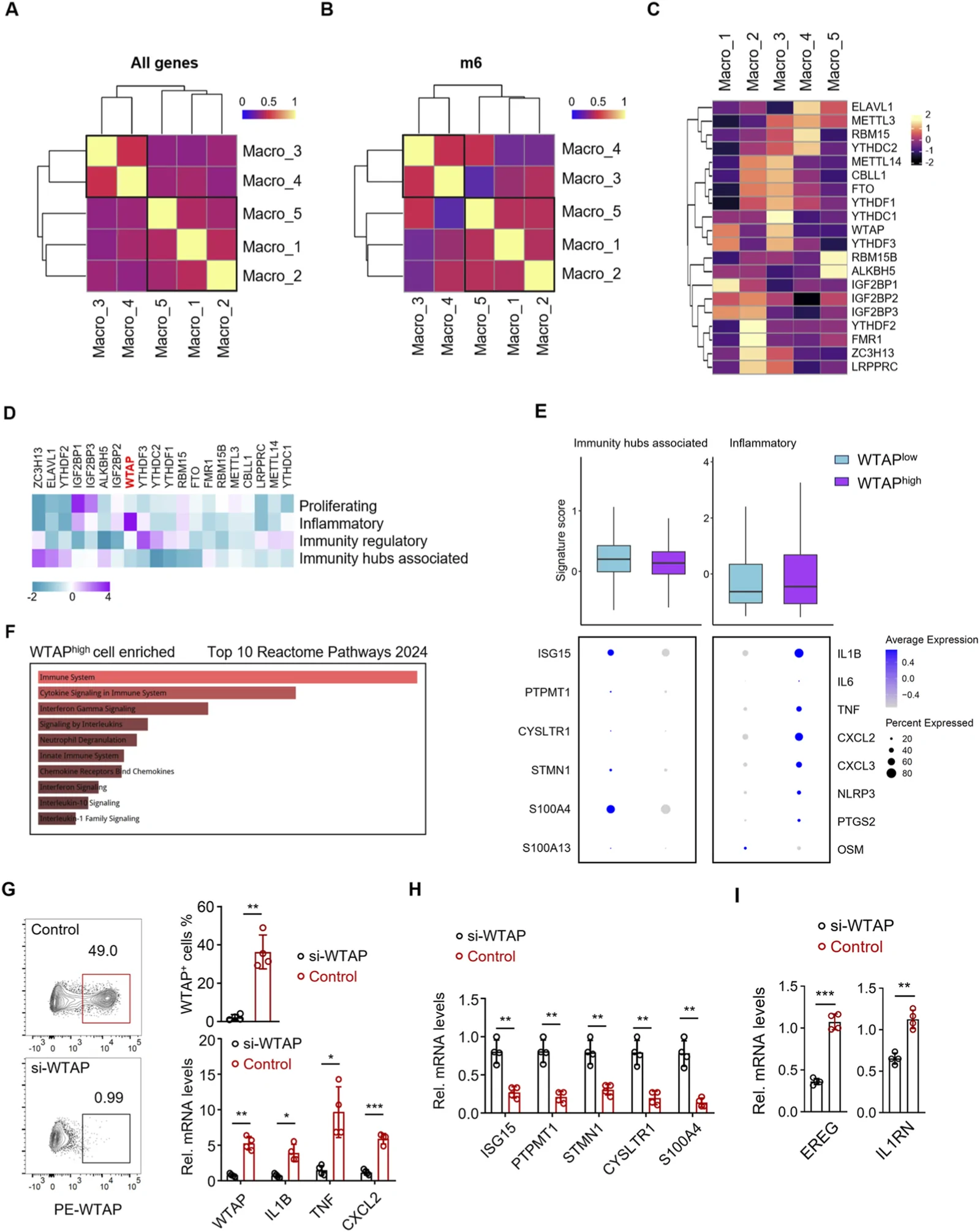
*WTAP* is associated with inflammatory reprogramming during macrophage state transitions. **(A)** Comparison of Unsupervised clustering results of Macro_1-Macro_5 based on all genes. **(B)** Comparison of unsupervised clustering of Macro_1-Macro_5 based on m^6^A-related regulatory genes. **(C)** Heatmap showing the expression of m^6^A-associated regulatory genes across distinct macrophage subsets. **(D)** Correlation analysis between *WTAP* expression and macrophage functional signatures, including immune regulatory, inflammatory, proliferation, and immunity hub-associated programs. **(E)** Comparison of functional signature scores between *WTAP* ^high^ and *WTAP* ^low^ macrophages, including inflammatory and immunity hub-associated programs. Scores are shown at the single-cell level to characterize cellular heterogeneity. **(F)** Reactome pathway enrichment analysis showing the top 10 enriched pathways in *WTAP* ^high^ macrophages. **(G)** Flow cytometric analysis of WTAP protein expression and qPCR analysis of *WTAP* knockdown efficiency and expression levels of representative pro-inflammatory genes (*TNF*, *IL1B*, *CXCL2*) in monocyte-derived macrophages following inflammatory stimulation in si-*WTAP* (n = 4) and control groups (n = 4). **(H)** qPCR analysis of immunity hub-associated genes (*ISG15*, *PTPMT1*, *STMN1*, *CYSLTR1*, *S100A4*) in monocyte-derived macrophages following *WTAP* knockdown (si-*WTAP*) (n = 4) compared with control (n = 4). **(I)** qPCR analysis of the *EREG* and *IL1RN* expression levels in monocyte-derived macrophages following *WTAP* knockdown (si-*WTAP*) (n = 4) compared with control (n = 4). *, *P* < 0.05; **, *P* < 0.01; ***, *P* < 0.001 (two-tailed paired Students’ t-test in **(G,I)**.

At the single-gene level, m^6^A regulators exhibited heterogeneous expression patterns across macrophage subsets (Figure 5C), supporting state-dependent regulatory specialization. Correlation analysis further identified *WTAP* as a candidate regulator associated with inflammatory programs, showing positive association with inflammatory programs and negative correlation with immunity hub-associated signatures (Figure 5D), suggesting a potential association with the inflammatory macrophage state. These observations suggest that *WTAP* may be associated with inflammatory macrophage states, potentially through epitranscriptomic mechanisms. To further characterize *WTAP*-associated functional states, macrophages were stratified based on WTAP expression. *WTAP* ^high^ macrophages exhibited reduced activity of immunity hub-associated gene programs, whereas inflammatory programs were elevated, indicating a shift toward a pro-inflammatory state (Figure 5E). Functional pathway enrichment analysis further demonstrated that *WTAP* ^high^ macrophages were significantly enriched in inflammatory and innate immune signaling pathways, including cytokine signaling, interferon-γ signaling, interleukin signaling, chemokine-mediated responses, neutrophil degranulation, and IL-1 family signaling (Figure 5F), supporting a coordinated activation of immune effector programs.

To functionally validate the role of *WTAP*, we established an *in vitro* monocyte-derived macrophage system and performed siRNA-mediated knockdown. Efficient silencing of *WTAP* was confirmed at the mRNA level (Figure 5G). Flow cytometric analysis further confirmed a significant reduction in the proportion of WTAP^+^ macrophages in si-*WTAP*-treated macrophages (Figure 5G), consistent with the mRNA results. Following LPS and IFN-γ stimulation, *WTAP*-depleted macrophages exhibited markedly reduced expression of canonical inflammatory genes, including *TNF*, *IL1B*, and *CXCL2* (Figure 5G). In contrast, genes associated with immunity hub-like programs, including *ISG15*, *PTPMT1*, *STMN1*, *CYSLTR1*, and *S100A4*, were relatively increased in *WTAP*-deficient cells (Figure 5H), indicating a partial shift away from a pro-inflammatory transcriptional state. Consistently, *WTAP* depletion significantly reduced the expression of *EREG* and *IL1RN* following inflammatory stimulation, further supporting an association between *WTAP* and the *EREG* ^+^ *IL1RN* ^+^ macrophage program (Figure 5I). Collectively, these results support *WTAP* as a potential regulator associated with macrophage inflammatory state transitions. Rather than merely reflecting lineage identity, m^6^A regulatory programs are associated with macrophage state transitions, and *WTAP* perturbation modulated the expression of key components of the *EREG* ^+^ *IL1RN* ^+^ macrophage program.

### The *EREG* ^+^ *IL1RN* ^+^ macrophage program is enriched in fetal membranes from clinical PPROM and associated with structural disruption

To further validate the clinical relevance of the conserved inflammatory macrophage subset, we analyzed bulk RNA-seq data from gestational age-matched fetal membrane tissues from gestational age-matched fetal membrane tissues from preterm labor (PTL) and preterm premature rupture of membranes (PPROM) cases (Underhill et al., 2024). Using macrophage-specific gene signatures, we quantified the relative activity of three programs (M_1-M_3) in these samples (Figures 6A,B). Among these, the M_3 program, corresponding to *EREG* ^*+*^ *IL1RN* ^*+*^ macrophages, was markedly elevated in PPROM membranes compared with PTL controls, providing independent clinical evidence that this inflammatory macrophage program is associated with PPROM. To assess the exploratory discriminatory ability of this signature, we evaluated its ability to distinguish PPROM from PTL cases. Among the three macrophage programs, M_3 exhibited the highest observed AUC for PPROM (AUC = 0.781, 95% CI: 0.45–0.99), compared with M_1 (AUC = 0.625, 95% CI: 0.33–0.92) and M_2 (AUC = 0.516, 95% CI: 0.19–0.83) (Figure 6C). These results suggest that elevated M_3 (*EREG* ^*+*^ *IL1RN* ^*+*^) macrophage activity is associated with PPROM and may have potential translational relevance. Given the small sample size and the postpartum collection of fetal membrane tissues, these findings should be interpreted as preliminary evidence of an association rather than as evidence for prospective prediction or identification of high-risk pregnancies. Collectively, these findings identify the *EREG* ^*+*^ *IL1RN* ^*+*^ macrophage program as a PPROM-associated inflammatory signature enriched in fetal membranes, while not establishing that the program represents a direct pathogenic mechanism of PPROM (Figure 6D).

**FIGURE 6:**
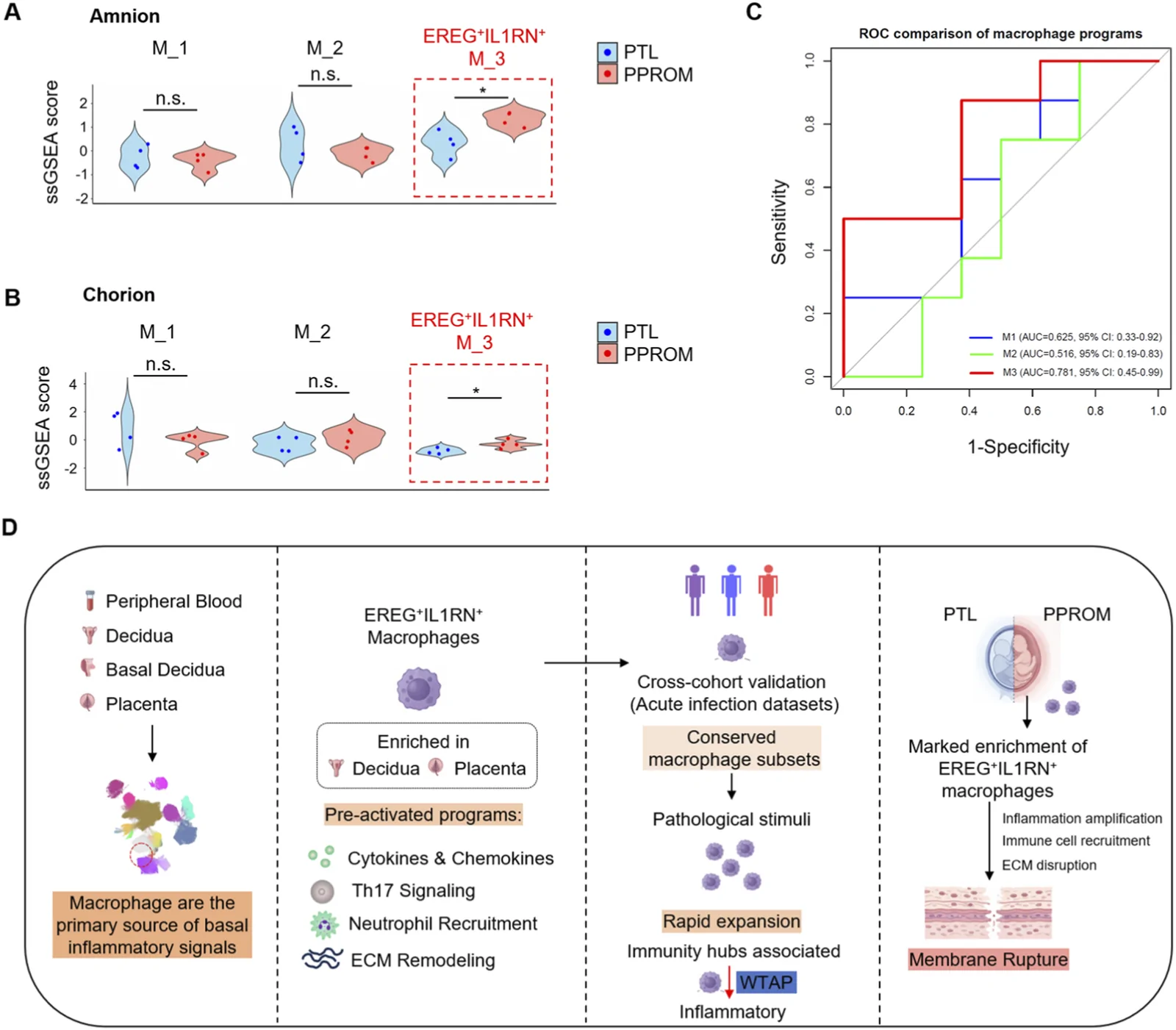
*EREG* ^*+*^ *IL1RN* ^*+*^ inflammatory macrophages are enriched in Amnion and Chorion of PPROM fetal membranes. **(A)** Violin plots showing macrophage-specific gene signature scores (M_1-M_3) in Amnion samples from gestational age-matched preterm labor (PTL) (n = 4) and preterm premature rupture of membranes (PPROM) (n = 4) cases. The M_3 program corresponding to *EREG* ^*+*^ *IL1RN* ^*+*^ inflammatory macrophages. **(B)** Violin plots showing macrophage-specific gene signature scores (M_1-M_3) in Chorion samples from gestational age-matched PTL (n = 4) and PPROM (n = 4) cases, highlighting the enrichment of the M_3 inflammatory macrophage program in PPROM. **(C)** ROC analysis was performed to evaluate the exploratory discriminatory capacity of the M_1, M_2, and M_3 macrophage signatures for distinguishing PPROM from PTL samples. **(D)** Schematic model illustrating the proposed role of *EREG* ^*+*^ *IL1RN* ^*+*^ inflammatory macrophages in PPROM pathogenesis. n. s., no significance; *p < 0.05 (two-tailed unpaired Students’ t-test in **(A,B)**.

## Discussion

Inflammation at the maternal-fetal interface is increasingly recognized as a dynamic process that coordinates immune surveillance, tissue remodeling, and host defense throughout pregnancy (Bezemer et al., 2024; Fell et al., 2025). While excessive inflammation is a well-established driver of adverse pregnancy outcomes, including PPROM(Neamtu et al., 2025), the cellular populations responsible for initiating and amplifying these responses remain incompletely defined. By integrating multiple single-cell, single-nucleus, spatial transcriptomic, and clinical transcriptomic datasets, we identified a conserved *EREG* ^*+*^ *IL1RN* ^*+*^ macrophages that occupies a central position within inflammatory networks at the maternal-fetal interface. Furthermore, we provide evidence that this macrophage state is dynamically amplified under inflammatory stimulation and is associated with increased activity in PPROM fetal membranes. Together, these findings support a model in which a pre-existing inflammatory macrophage program contributes to the transition from physiological immune homeostasis to pathological inflammatory activation.

Recent single-cell studies have revealed substantial heterogeneity among maternal-fetal interface macrophages, challenging the traditional M1/M2 framework and suggesting the existence of specialized macrophage states adapted to distinct tissue niches (Vondra et al., 2023). Consistent with this concept, our analysis resolved multiple macrophage subsets with distinct transcriptional identities and tissue distributions. Among these populations, *EREG* ^+^ *IL1RN* ^+^ macrophages displayed the strongest inflammatory signatures and were associated with an inflammatory state along the inferred macrophage transcriptional continuum. Notably, this subset co-expressed inflammatory mediators, including *PTGS2* and *NLRP3*, together with regulatory molecules such as *IL1RN* and *EREG*. The co-expression of *EREG*, a growth factor implicated in tissue repair and inflammatory regulation (Riese and Cullum, 2014), alongside IL1RN, the endogenous IL-1 receptor antagonist that constrains excessive inflammation, suggests a tightly regulated inflammatory state rather than uncontrolled immune activation. Rather than representing a purely pro-inflammatory population, this transcriptional configuration suggests a highly responsive macrophage state that integrates inflammatory activation with compensatory regulatory mechanisms. Such a balanced architecture may enable rapid responses to environmental perturbations while limiting uncontrolled immune activation within the maternal-fetal niche.

A major finding of this study is the remarkable conservation of the *EREG* ^*+*^ *IL1RN* ^*+*^ macrophage program across independent datasets and experimental contexts. The consistent identification of this population in integrated single-cell atlases, independent single-nucleus datasets, and spatial transcriptomic analyses argues against a dataset-specific phenomenon and instead supports the existence of a conserved inflammatory macrophage state at the maternal-fetal interface. Moreover, the expansion of transcriptionally similar macrophages in placental infection models involving diverse pathogens suggests that this program represents a generalizable inflammatory response rather than a pathogen-specific adaptation. These observations support a “baseline-to-pathological activation” framework, in which a conserved inflammatory macrophage compartment exists under homeostatic conditions and becomes transcriptionally amplified in response to inflammatory challenges.

Functional analyses further suggest that *EREG* ^*+*^ *IL1RN* ^*+*^ macrophages may act as important coordinators of inflammatory amplification. In addition to elevated cytokine and chemokine programs, this subset displayed enrichment of neutrophil recruitment, Th17-associated signaling, and extracellular matrix remodeling pathways. Neutrophil infiltration and Th17-mediated immune responses have been increasingly implicated in tissue injury and adverse pregnancy outcomes. Concurrent activation of these programs suggests that *EREG* ^*+*^ *IL1RN* ^*+*^ macrophages may function as cellular hubs linking inflammatory sensing to downstream effector responses. The enrichment of extracellular matrix remodeling signatures further indicates potential involvement in tissue structural changes, consistent with the established role of matrix metalloproteinase activation and collagen degradation in fetal membrane weakening (Moore et al., 2006; Strauss, 2013). These processes have previously been implicated in inflammatory tissue injury and adverse pregnancy outcomes. Therefore, *EREG* ^*+*^ *IL1RN* ^*+*^ macrophages may represent a cellular interface through which inflammatory sensing is coupled to downstream immune recruitment and tissue remodeling. Although the precise mechanisms remain to be determined, our findings position this macrophage state as a potential mediator linking immune activation to alterations in tissue homeostasis at the maternal-fetal interface.

Beyond defining a conserved inflammatory macrophage state, our study identifies *WTAP* as a potential epitranscriptomic regulator of macrophage functional plasticity. Emerging evidence indicates that m^6^A RNA modification plays a critical role in shaping immune-cell activation and inflammatory responses (Luo et al., 2021; Shulman and Stern-Ginossar, 2020), yet its role in maternal-fetal interface macrophages has remained largely unexplored. We found that macrophage organization reconstructed using m^6^A regulators partially recapitulated transcriptome-defined functional states and identified *WTAP* as a regulator closely associated with inflammatory macrophage programs. Functional perturbation experiments further demonstrated that *WTAP* depletion attenuated inflammatory gene expression while promoting immunity hub-associated transcriptional features. Notably, *WTAP* depletion also reduced the expression of *EREG* and *IL1RN* following inflammatory stimulation, providing experimental support for an association between *WTAP* activity and key components of the *EREG* ^+^ *IL1RN* ^+^ macrophage program. Together, these findings implicate *WTAP* as a potential regulator of inflammatory macrophage remodeling, while the specific m^6^A-dependent molecular mechanisms and downstream targets remain to be determined. Although the direct molecular targets remain to be identified, our results highlight m^6^A-associated regulation as a potential layer contributing to macrophage state transitions at the maternal-fetal interface.

The enrichment of the *EREG* ^*+*^ *IL1RN* ^*+*^ macrophage program in clinical PPROM samples further supports its potential pathological relevance (Underhill et al., 2024). Among the macrophage-derived signatures evaluated in this study, the *EREG* ^*+*^ *IL1RN* ^*+*^ program demonstrated the strongest ability to distinguish PPROM from PTL samples, suggesting a specific association with membrane rupture-related pathology rather than preterm labor alone. However, the ROC analysis was based on only four PPROM and four PTL cases, and the wide confidence intervals indicate substantial uncertainty in the estimated discriminatory capacity. Therefore, these findings should be considered preliminary evidence of association and discrimination rather than evidence of prospective clinical prediction or risk stratification. Given the concurrent enrichment of inflammatory and extracellular matrix remodeling programs, these observations raise the possibility that activation of this macrophage state contributes to the inflammatory microenvironment associated with fetal membrane dysfunction. While causal relationships cannot be established from transcriptomic analyses alone, our findings provide a cellular framework linking immune activation with tissue-level alterations observed in PPROM.

Several limitations should be acknowledged. First, this study relies primarily on integrative analyses of publicly available transcriptomic datasets, and independent validation in larger prospective cohorts will be required. In particular, the PPROM analysis included only four PPROM and four PTL cases, resulting in wide confidence intervals around the ROC estimates. The ROC findings should therefore be regarded as exploratory and cannot establish prospective prediction of PPROM risk or clinical risk stratification. Larger, independent cohorts will be required to determine the reproducibility and potential clinical relevance of the M_3 signature. The macrophage atlas and inflammatory stimulation model were primarily derived from early pregnancy tissues, whereas PPROM typically occurs in mid-to-late pregnancy. Although the *EREG* ^+^ *IL1RN* ^+^ program was also enriched in gestational age-matched PPROM fetal membrane samples, this finding remains preliminary and does not establish a direct pathogenic role in PPROM. Second, the biological functions of *EREG* ^*+*^ *IL1RN* ^*+*^ macrophages were inferred predominantly from transcriptomic analyses, and direct protein-level validation in clinical fetal membrane tissues was not performed. In particular, multiplex IF, IHC, or RNAscope validation of *EREG* and *IL1RN* together with macrophage markers in independent PPROM and PTL fetal membrane specimens will be important to further confirm the cellular localization and protein-level characteristics of this macrophage program. Additional experimental studies are needed to determine their direct contributions to membrane remodeling and pregnancy outcomes. Third, although *WTAP* depletion reduced the expression of *EREG* and *IL1RN* in monocyte-derived macrophages, these functional experiments were performed in an *in vitro* model rather than macrophages directly isolated from the maternal-fetal interface. Therefore, this system may not fully recapitulate the tissue-specific microenvironment in which the *EREG* ^+^ *IL1RN* ^+^ macrophage program was identified. Further studies using primary maternal-fetal interface macrophages or complementary *in vivo* models will be required to determine the tissue-specific role of *WTAP* in this macrophage state. In addition, the downstream m^6^A-modified targets and molecular mechanisms through which *WTAP* influences macrophage inflammatory states remain to be determined.

In summary, we identify *EREG* ^*+*^ *IL1RN* ^*+*^ macrophages as a conserved inflammatory macrophage program at the maternal–fetal interface and provide evidence that this state is amplified under inflammatory conditions and associated with PPROM. We further implicate *WTAP* as a potential epitranscriptomic regulator associated with inflammatory macrophage reprogramming. Together, these findings offer new insight into the cellular and regulatory basis of inflammatory activation during pregnancy and establish a framework for investigating macrophage-targeted strategies in inflammation-associated pregnancy complications.

## Data Availability

The datasets presented in this study can be found in online repositories. The names of the repository/repositories and accession number(s) can be found in the article/Supplementary Material.
